# Clinical service desires of medical cannabis patients

**DOI:** 10.1186/1477-7517-9-12

**Published:** 2012-03-13

**Authors:** Jennifer L Janichek, Amanda Reiman

**Affiliations:** 1 Institute for Metropolitan Affairs, Roosevelt University, Chicago, USA; 2 University of California, Berkeley, USA

**Keywords:** Medical cannabis patients, Harm reduction, Cannabis dispensaries, Substance use, Substance misuse

## Abstract

**Background:**

Medical cannabis dispensaries following the social or hybrid model offer supplementary holistic services in addition to dispensing medical cannabis. Historically, alternative physical health services have been the norm for these dispensaries, including services such as yoga, acupuncture, or chiropractor visits. A clinical service dearth remains for medical cannabis patients seeking substance use, misuse, dependence, and mental health services. This study examined patient desires for various clinical services and level of willingness to participate in specific clinical services.

**Methods:**

Anonymous survey data (N = 303) were collected at Harborside Health Center (HHC), a medical cannabis dispensary in Oakland, CA. The sample was 70% male, 48% Caucasian and 21% African American. The mean male age was 38 years old and female mean age was 30. Sixty two percent of the male participants and 44% of the female participants are single. Sixteen percent of the population reported having a domestic partner. Forty six percent of the participants are employed full time, 41% have completed at least some college, and 49% make less than $40,000 a year.

**Results:**

A significant portion of the sample, 62%, indicated a desire to participate in free clinical services at HHC, 34% would like more information about substances and use, and 41% want to learn more about reducing harms from substance use. About one quarter of the participants marked "would" or "likely would" participate in individual services such as consultation. Approximately 20% indicated "would" or "likely would" participate in psycho-educational forums, harm reduction information sharing sessions, online support groups, and coping, life, and social skills group. There was little interest in traditional NA/AA 12-step groups or adapted 12-step groups.

**Conclusions:**

Desired clinical services can be qualified as a combination of harm reduction, educational, skills-based, peer support and therapeutic individual and group services. Results suggest that medical cannabis patients seek more information about various substances, including cannabis. Dispensaries can help to decrease gaps in substance education and clinical services and fulfill unmet clinical desires. More research is necessary in additional medical cannabis dispensaries in different geographic settings with different service delivery models.

## Background

Individuals who use medical cannabis for therapeutic purposes are a diverse, evolving, and a generally understudied medical cohort. An increasing shift from the use of "mainstream" medications, namely pharmaceuticals, to the use of "natural" or alternative medications has been observed [1,2]. The most noted reasons for the transition from synthetic medication to medical cannabis include: perception that conventional medication was problematic and/or ineffective, cannabis produced fewer side effects (or drawbacks), and cannabis resulted in better symptom control and increased relief when compared to previous treatments and medications [2-4]. Cannabis is viewed as more "natural," when compared to manufactured synthetic medication, which is appealing for some medication consumers [2].

In select states, such as California, Colorado and Maine, there is an emergence of secure and reliable access to cannabis through medical cannabis dispensaries, although these organizations remain unlicensed and illegal at the federal level and are still subject to federal enforcement efforts. Also known as patient collectives, buyers' clubs, or wellness centers, these community based health facilities provide cannabis to individuals who have obtained a doctor recommendation for its medical use. These facilities enable medical cannabis patients to safely purchase medication and many facilities offer a range of products from dried cannabis flowers and cannabis concentrates to various forms of topical and edible cannabis products [5].

Medical cannabis health centers may provide an opportune therapeutic setting to offer other clinical services, such as substance misuse and related mental health clinical services [6]. In a study by Reiman (2008), sixty six percent of the patients surveyed reported utilization of the holistic services in San Francisco Bay Area medical cannabis facilities [5]. Currently, various social and physical services are offered at dispensaries such as massage therapy, nutritional and herbal consultations, peer groups, and acupuncture are offered at some dispensaries [5]. However, few dispensaries offer clinical services related to substance use, misuse, dependence, and mental health services.

The National Survey on Drug Use and Health demonstrates the longstanding unmet clinical service need of individuals seeking substance misuse, addiction, and mental health services in the United States. The most recent survey in 2009 shows that almost 9 percent of the U.S. population, or 22.5 million people, were categorized to fit the substance abuse or dependence diagnosis, but 1.7 percent, or 4.3 million people, received treatment for substance misuse [7]. The poor availability of treatment services is referred to as a service gap for the general population, meaning more individuals desire services than there are services available.

Restrictive entrance parameters and program rules such as a "clean" drug tests (meaning free from all substances), are often a condition for receiving services [8]. Other noted barriers include: the high cost of private treatment services, lack of insurance, and not wanting to be(or failing to be) abstinent, which is required in many clinical settings [9]. Given the abstinence demands of many treatment programs, having a medical cannabis patient status may in itself be a barrier to receiving desired services for mental health and substance misuse and addiction. The need for integrated clinical services at cannabis health centers can also driven by a lack of formal substance health education, resulting in ill informed substance consumers. In addition, because cannabis is different than other traditional medications, patients might have less traditional medical guidance, and little empirical, applied data to refer to about safe and effective medical cannabis use. Substance use stigma also continues to be a barrier for individuals seeking treatment for substance misuse. Clinical services integrated within medical cannabis health centers may help close service gaps and reduce service barriers for those seeking services.

One promising area in the nexus of medical cannabis and substance abuse treatment is the potential of cannabis to serve as a tool to assist a person to "exit" or relinquish and abstain from another more harmful substance [10]. Individuals who use cannabis to assist in reducing or abstaining from the use of other substances, either viewed their substitution as very effective or effective in curbing the harms associated with addiction [11]. Substance substitution studies show a large percentage of individuals use medical cannabis as a substitute for alcohol (40%), for illicit substances (26%), and prescription drugs (65.8%) [5,12]. Substance substitution with cannabis has been related to fewer side effects and increased symptom management as compared to other substances [5]. In a recent study of medical cannabis patient profiles revealed that 51 percent report using cannabis as a substitute for prescription medication, supporting previous research on cannabis substitution [13].

Cannabis may also serve as a useful therapeutic alternative for individuals seeking addiction and mental health symptom management [14-16]. A meta-analysis on the subjective effects of cannabis found that the most frequently reported effects were: improved mood (i.e., feeling good, happy, content), enhanced relaxation, increased insight into self and others, and improved perceptions. Of the reviewed close-ended studies, improved thinking, increased concentration, and increased relaxation were frequently endorsed. Authors note individual variations due to substance tolerance, setting, and cognitive set [17]. Surveys from Australia and Germany and exploratory studies in Canada and the United states find that 12 to 56 percent of medical cannabis users report use for relief of symptoms of depression, 6 percent for the relief of anxiety symptoms, and 6 percent for relief from other psychological disorders [3,18-20].

Recent findings show that current medical cannabis consumers might fare as well or better than other cohorts who enter addiction treatment. Preliminary findings indicate that medical cannabis use does not interfere negatively with traditional substance addiction treatment; in fact medical cannabis patients had a higher treatment completion rate when compared to other individuals in treatment [21]. Additionally, two studies demonstrate that intermittent cannabis users show superior retention in naltrexone opiate treatment [22,23].

This exploratory study asks the question: What is the level of desire for and willingness to participate in clinical mental health and substance misuse services among medical cannabis patients? This study attempted to capture the "patient voice" to inform the creation of a harm reduction clinical services program at a hybrid model dispensary in Oakland, CA.

## Methods

### Sample

Survey participants were patient members of Harborside Health Center (HHC), a medical cannabis collective located in Oakland, CA. To date the HHC patient membership is approximately 45,000 individuals. The survey sample included 303 medical cannabis patients between the ages of 18 and 75. The mean age was 38 years old. The sample was 70.3% male, 48.2% Caucasian, and 20.8% African American. Fifty six percent were single and 7.6% report to have a domestic partner. Sixty two percent are employed, 15% are on disability income, and 37% have attained a bachelor or graduate degree. Fifty one percent of the sample earned over $40,000 annually.

## Materials

Researchers created the survey and included direct sections from a Reiman 2009 survey of patients accessing cannabis through Berkeley Patients Group (N = 350). The survey contained a total of 48 questions including 14 yes or no questions, 18 likert scale questions, 11 multiple choice questions, and 5 open-ended questions. The domains of interest included: basic patient demographic information; present use of cannabis, alcohol, tobacco, and other substances and use in past 30 days; prior experiences with clinical services and possible barriers encountered with services; patient desire to change substance use related behaviors; desire for more information about substances and substance use; and level of willingness to participate in particular clinical services at HHC.

### Procedures

The lead researcher collected data at the dispensary during the hours of 11 am to 8 pm over a 4-week time period, including weekdays and weekends. After the mandatory patient check-in, front desk staff asked every patient to participate in an anonymous survey. If the individual was willing to participate, the researcher briefly explained the purpose of the survey, confidentiality and risks of participation, and the right to refuse or stop the survey. Informed consent information was also provided in written form. Harborside Health Center funded this survey as part of a program development proposal. Data were analyzed and frequencies were calculated.

## Results

### Medical cannabis, alcohol, tobacco, and other substance use

Eighty eight percent of the sample reported daily use of medical cannabis and 28% of the sample consume 3 to 5 grams of cannabis per week. Fifty five percent currently drink alcohol and the average number of drinking days per month was 8.95. Twenty five percent report tobacco use and 15% of the sample have used another substance within the past 30 days. Of the individuals who used another substance other than cannabis, alcohol, or tobacco (N = 46), pharmaceuticals account for 58.70% of the substances used. Furthermore, opiate-derived pharmaceuticals account for 39.13% of the substances used (Table 1, 2).

**Table 1:** Medical Cannabis, Alcohol, and Tobacco Usage Within Past 30 Days Reported by Medical Cannabis Patients (N = 303)

Substance	Reported Usage	Percent of Survey Participants
Medical Cannabis	303	100.00%

Alcohol	166	54.79%

Tobacco	76	25.08%

**Table 2:** Other Substance Usage Within Past 30 Days Reported by Medical Cannabis Patients (N = 303)

Substance	Reported Usage	Percent of Survey Participants
Opioid Pharmaceuticals	18	5.95%

Hallucinogens	11	3.63%

MDMA/Ecstasy	10	3.30%

Cocaine/Crack Cocaine	7	2.31%

Benzodiazepine Pharmaceuticals	6	1.98%

Other Substances	5	1.65%

Stimulant Pharmaceuticals	3	0.99%

Methamphetamine	2	0.66%

Heroin	0	0.00%

Opioid pharmaceuticals may include Vicodin, Percocet, Codeine, Hydrocodone, Oxycodone, Buprenorphine, Fentanyl, etc. Hallucinogens may include psilocybin mushrooms, mescaline, LSD, etc. Benzodiazepine pharmaceuticals may include Valium, Ativan, Xanax, Klonopin, Rohypnol, etc. Stimulant pharmaceuticals may include Concerta, Adderall, Dexedrine, Ritalin, etc.

### Substance misuse and addiction treatment

Four percent of the sample had previously attended treatment services for alcohol dependence, 2% currently attend 12-step support groups, and 10% have received treatment for substance-related problems in the past. Five percent had previously been diagnosed with a substance dependence disorder. Eight percent had wanted to attend treatment for substance misuse or dependence but did not participate. Of those participants who wanted to attend treatment but did not attend, the following reasons were most noted: not ready for change (3%), couldn't afford it (2%), program philosophy (2%), required abstinence (2%), drug testing policies (2%), and none available (2%).

### Desire to change substance use behaviors

Seventy five percent of the current tobacco smokers "probably" to "definitely" want to abate or stop the use of tobacco. Seventy three percent of the alcohol consumers "probably do not" and "definitely do not" want to reduce or stop use of alcohol. Fifty eight percent of the individuals who used another substance in the last 30 days "probably do not" and "definitely do not" want to reduce or stop use of the specified substance (Table 3).

**Table 3:** Desire to Reduce or Stop the Use of Tobacco, Alcohol and Other Drugs Reported by Medical Cannabis Patients (N = 303)

Substance	Definitely Not	Probably Not	Maybe	Probably	Definitely
**Tobacco**	2	6	12	16	41

**Alcohol**	66	46	24	9	10

**Other Substances**	46	20	9	5	7

### Desire for clinical services at a medical cannabis health center

Thirty four percent reported wanting want access to more factual information about substances and substance use and 41% want to learn more about how to reduce harms from substance use. Sixty two percent said they would participate in free clinical services at Harborside Health Center, if the services were offered. Twenty seven percent of the survey participants indicated, "I will or I likely would" participate in brief individual counseling and twenty five percent for individual consultation, assessment, and referral. Twenty percent marked "I will or I likely would" participate in mental health forums, 19% in substance education sessions, 19% in skills groups, and 17% in an online therapeutic support group. Less than 10% of the participants would or likely would participate in recovery maintenance groups, NA/AA 12-steps groups, and modified NA/AA 12-step groups (Table 4).

**Table 4:** Medical Cannabis Patient Desire to Participate, by Clinical Service Type (N = 303)

Clinical Service Type	I WILL/ I Likely Would Participate		I May or May Not Participate		I Likely Would Not/ I Would NOT Participate	
	**Number**	**Percent**	**Number**	**Percent**	**Number**	**Percent**

Brief Individual Counseling	81	27%	67	22%	155	51%

Individual Assessment	75	25%	70	23%	158	53%

Medication Supports	69	23%	64	21%	170	56%

Combined Individual and Group Services	62	21%	62	20%	179	59%

Psycho-educational Forums	60	20%	61	20%	182	60%

Harm Reduction Sessions	57	19%	56	18%	190	63%

Coping, Life and Social Skills Group	56	19%	64	21%	183	61%

Online Therapeutic Support Group	51	17%	72	24%	180	60%

Tobacco Cessation Group	46	16%	44	15%	213	70%

Group Psychotherapy	41	14%	59	19%	203	67%

Rap Group	41	14%	55	18%	207	68%

Recovery Maintenance Group	26	9%	52	17%	225	75%

Adapted NA/AA, Harm Reduction Group	19	7%	55	18%	229	76%

NA/AA 12-Step Group	18	6%	49	16%	236	78%

Adapted NA/AA, Non-Religious Group	17	6%	57	19%	229	76%

## Discussion

To date there are no studies asking medical cannabis patients, who purchase medicine from a medical cannabis dispensary, about desires for dispensary based clinical services. Prior to the introduction of potential clinical services, at HHC a supplementary holistic care center arose from patient demand. The clinical services were described as an additional component to the holistic care program. When patients were asked about harm reduction services a marked desire for integrated clinical services was reported. A large portion of the patients sought more information about cannabis as well as more information about other substances. This may speak to the poor success rate of formal drug education programs provided in schools and oversaturation of information on the internet.

There was variance in type of clinical service desired. Two hundred and twenty five of the survey participants "would" or "most likely would" seek individual services including individual counseling, individual assessment and referral, and individual consultation with a psychiatrist for medication supports. One hundred and seventy three of the participants indicated desire for life skills groups such as problem solving and decision making, anger management skills, stress management, coping skills, and relational and communication skills. The high saturation of group services available in the community may be one explanation as to why group services, particularly AA/NA or 12-step groups, were not desired by the survey participants. A negative reputation, zero-tolerance for cannabis, and a lack of need may be reasoning for the participants' low level of desire to participate in this type of clinical service.

Results indicate that the majority of the medical cannabis patients do not use, misuse, or become addicted to illegal substances. This is evident in the use data, as well as the current and past treatment data. A small cohort of the medical cannabis patients currently (3.63%) or have in the past (14.52%) attended a treatment program for substance misuse or addiction, however the majority of patients have not recently used an illegal substance (85%), have never sought treatment, and did not find treatment necessary for any substance including cannabis. Medical cannabis patients who use tobacco were the only group of individuals dissatisfied with their behavior, indicating a desire to reduce or quit the use of this substance. The gateway theory theorizes that an individual's use of cannabis will lead the individual into use, misuse, and addiction of other "hard" substances such as cocaine and heroin. Many studies refute this theory including this exploratory study [24-27].

There is no standardized data collection protocol or universal data system at medical cannabis health centers that catalogue patient characteristics, therefore representation of HHC patient membership (N = ~45,000 individuals) is unclear. Recent descriptive studies of medical cannabis patients reveal that survey participant characteristics in this study such as age, gender, and employment status resemble those in the California medical cannabis patient cohort [4,5,13]. In addition, the surveyed patient population by and large mimics the general population characteristics of the surrounding area. Similar to the sample characteristics, the median age is 39 in the San-Francisco-Oakland-Fremont metropolitan statistical area [28]. When compared to census data a slight overrepresentation of males and single individuals in the sample may have occurred. There may be sample characteristic restrictions because the survey was administered at one medical cannabis dispensary over a limited time period. Since little is known about the frequency of patient visits, a 4-week data collection window may have missed a subsection of patients. Extremely ill patients may have been unable to participate because of mobility limitations or inability to frequent the health center. Additionally, this survey does not account for the individuals who use cannabis medicinally without a physician recommendation and therefore are not patronizing medical cannabis dispensaries for medication. Last, self-selection may be present as related to the past illegal status of cannabis and societal stigma of substance use.

While survey data was used for applied purposes, findings show that medical cannabis patients desire clinical services, qualified as a combination of harm reduction, educational, skills-based, peer support and therapeutic individual and group services. Medical cannabis dispensaries can help to decrease commonplace clinical service gaps and fulfill unmet clinical desires. More research is necessary in additional medical cannabis dispensaries in other jurisdictions and with potentially different service delivery models to help uncover the characteristics of this once abjured cohort.

## Competing interests

Jennifer Janichek is compensated for her position as a harm reduction counselor at Harborside Health Center. Amanda Reiman is compensated for her position as Director of Research at Berkeley Patients Group.

## Authors' contributions

JJ conceived the study and JJ and AR contributed to the development of the data collection instrument, sampling strategy and study protocol. JJ carried out the data collection and data analysis. JJ and AR drafted the manuscript, read and approved the final manuscript.

## References

[B1] Northcott H Bolaria BS, Dickinson HD Alternative health care in Canada Health, illness and health care in Canada 1994 2 Toronto: Harcourt Brace 487 503

[B2] Coomber R Oliver M Morris C Using cannabis therapeutically in the UK: A qualitative analysis J Drug Issues 2003 33 2 325 356 10.1177/002204260303300204

[B3] Swift W Gates P Dillon P Survey of Australians using cannabis for medical purposes Harm Reduction Journal 2005 2 18 10.1186/1477-7517-2-18 PMC1262744 16202145

[B4] Reiman AE Medical cannabis patients: Patient profiles and health care utilization patterns Complement Heal Pract Rev 2007 12 1 31 50 10.1177/1533210107301834

[B5] Reiman AE Self-efficacy, social support and service integration at medical cannabis facilities in the San Francisco Bay Area of California Health Soc Care Community 2008 16 1 31 41 18181813 10.1111/j.1365-2524.2007.00722.x

[B6] Feldman HW Mandel J Providing medical marijuana: The importance of cannabis clubs J Psychoactive Drugs Spec Issue Marijuana Millenn Med Soc Implications 1998 30 2 179 186 10.1080/02791072.1998.10399688 9692380

[B7] U.S. Department of Health and Human Services, Substance Abuse and Mental Health Services Administration, Office of Applied Studies, 2009 National Survey on Drug Use and Health: National Findings http://oas.samhsa.gov/NSDUH/2k8NSDUH/2k8results.cfm#TopOfPage Retrieved June 25, 2010

[B8] Cunningham JA Sobell LC Sobell MB Agrawal S Toneatto T Barriers to treatment: Why alcohol and drug abusers delay or never seek treatment Addict Behav 1993 18 347 353 10.1016/0306-4603(93)90036-9 8393611

[B9] U.S. Department of Health and Human Services, Substance Abuse and Mental Health Services Administration, Office of Applied Studies, 2009 National Survey on Drug Use and Health: National Findings http://oas.samhsa.gov/nsduh/2k8nsduh/2k8Results.cfm#Ch7 Retrieved June 25, 2010

[B10] Newman R "Maintenance" treatment of addiction: To whose credit, and why it matters Int J Drug Pol 2009 20 1 3 10.1016/j.drugpo.2008.07.001 18775657

[B11] Mikuriya T Cannabis as a substitute for alcohol: A harm-reduction approach J Cannabis Therapeut 2004 4 1 79 93 10.1300/J175v04n01_04

[B12] Reiman AE Cannabis as a substitute for alcohol and other drugs Harm Reduction Journal 2009 6 35 10.1186/1477-7517-6-35 PMC2795734 19958538

[B13] Reinarman C Nunberg H Lanthier F Heddleston T Who are medical marijuana patients? Population characteristics from nine California assessment clinics J Psychoactive Drugs 2011 43 2 128 135 10.1080/02791072.2011.587700 21858958

[B14] Zuardi A Crippa J Hallak J Moreira F Guimaraes F Cannabidiol, a cannabis sativa constituent, as an antipsychotic drug Braz J Med Biol Res 2005 9 421 429 10.1590/s0100-879x2006000400001 16612464

[B15] Di Marzo V Bifulco M De Petrocellis L The endocannabinoid system and its therapeutic exploitation Nat Rev Drug Discov 2004 9 771 784 15340387 10.1038/nrd1495

[B16] Ashton C Moore P Gallager P Young A Cannabinoids in bipolar affective disorder: A review and discussion of their therapeutic potential J Psychopharmacol 2005 19 293 300 10.1177/0269881105051541 15888515

[B17] Green B Kavanagh D Young R Being stoned: A review of self-reported cannabis effects Drug Alcohol Rev 2003 22 4 453 460 10.1080/09595230310001613976 14660135

[B18] Schnelle M Grotenhermen F Reif M Gorter R Results of a standardized survey on the medical use of cannabis in the German-speaking area Compl Med 1999 3 28 36 10.1159/000057154 10575286

[B19] Ogborne AC Smart RG Weber T Birchmore-Timney C Who is using cannabis as a medicine and why: An exploratory study J Psychoactive Drugs 2000 32 4 435 443 10.1080/02791072.2000.10400245 11210205

[B20] Hathaway AD Rossiter K Medical marijuana, community building, and Canada's compassionate societies Contemp Justice Rev 2007 10 3 283 296 10.1080/10282580701526088

[B21] Swartz R Medical marijuana users in substance abuse treatment Harm Reduction Journal 2010 7 3 10.1186/1477-7517-7-3 PMC2848643 20202221

[B22] Church SH Rothenberg JL Sullivan MA Bornstein G Nunes EV Concurrent substance use and outcome in combined behavioral and naltrexone therapy for opiate dependence Am J Drug Alcohol Abuse 2001 27 441 452 10.1081/ADA-100104511 11506261

[B23] Raby WN Carpenter KM Rothenberg J Brooks AC Jiang H Sullivan M Bisaga A Comer S Nunes EV Intermittent marijuana use is associated with improved retention in naltrexone treatment for opiate-dependence Am J Addict 2009 18 301 308 10.1080/10550490902927785 19444734 PMC2753886

[B24] Morral A McCaffrey D Paddock S Reassessing the Marijuana Gateway Effect Addiction 2002 97 1493 1504 10.1046/j.1360-0443.2002.00280.x 12472629

[B25] O'Connell T Bou-Mater C Long term marijuana users seeking medical cannabis in California (2001-2007): Demographics, social characteristics, patterns of cannabis and other drug use of 4117 applications Harm Reduction Journal 2007 4 16 10.1186/1477-7517-4-16 PMC2175501 17980043

[B26] Tarter R Vanyukoy M Kirisci L Reynolds M Clark D Predictors of marijuana use in adolescents before and after licit drug use: Examination of the gateway hypothesis Am J Psychiatr 2006 63 12 2134 2140 17151165 10.1176/ajp.2006.163.12.2134

[B27] Van Gundy K Rebellon C Life-course perspective on the "Gateway Hypothesis J Health Soc Behav 2010 51 3 244 259 10.1177/0022146510378238 20943588

[B28] American Community Survey (2008 1 year estimates) Selected Population Profile in the United States, San Francisco-Oakland-Fremont, CA Metropolitan Statistical Area http://factfinder.census.gov/servlet/ADPTable?_bm=y&-geo_id = 31000US41860&-context=adp&-ds_name=ACS_2008_1YR_G00_&-tree_id = 308&-_lang=en&-_caller=geoselect&-format= Retrieved June 25, 2010

